# New Lectureships in the University of Pennsylvania

**Published:** 1865-11

**Authors:** 


### New Lectureships in the University of Pennsylvania.

The following statement in reference to this liberal act of Dr. Wood is taken from the *Medical and Surgical Reporter* of July 8th, 1865:

"Publicity having been given to the proposed endowment of Lectureships in the Medical Department of the University of Pennsylvania, we will state the facts as briefly as possible, so far as they have transpired.

"The Trustees of the University of Pennsylvania, through the liberality of the distinguished Emeritus Professor of the Theory and Practice of Medicine in that institution, Dr. George B. Wood, as we are informed, have devoted the sum of $50,000 to the endowment of lectureships in the University on the following subjects: 1. Zoology and Comparative Anatomy; 2. Botany; 3. Mineralogy and Geology; 4. Hygiene; 5. Medical Jurisprudence, including Toxicology. We have heard the names of competent gentlemen mentioned in connection with each of the chairs, but do not as yet feel authorized to give publicity to them. The appointments will be made in November next, and the first course of lectures will be given the ensuing spring. Each lecturer is to receive a salary of $500 from the fund, and also all fees that may accrue from the sale of tickets, the fee not to exceed $10, and regular matriculants and alumni to be admitted free.

"This liberal action of Dr. Wood is entirely in keeping with his well-known views on the subject of medical progress. He has always been a friend of thorough education, and when a teacher and professor, always insisted on a close application to his studies on the part of the student, and a proper qualification for the discharge of the responsible duties of the physician on the part of the graduate.

"In no better way could he have shown his love of, and confidence in, the science of medicine, and his attachment to his Alma Mater, the venerable University of Pennsylvania, than in the establishment of these lectureships. The devotion of a well-earned fortune to so high, honorable and ennobling a purpose as the advancement of medical education, would serve to immortalize the name of this distinguished friend of medical progress, even if he had done nothing more to secure to him so eminent a position."
*American Journal of Pharmacy.—Boston Medical Journal.*

